# Inclusion of Pineapple By-Products as Natural Antioxidant Sources in Diets for European Sea Bass (*Dicentrarchus labrax*)

**DOI:** 10.3390/antiox14030333

**Published:** 2025-03-11

**Authors:** Ricardo Pereira, Ana Basto, Manuela Pintado, Luisa M. P. Valente, Cristina Velasco

**Affiliations:** 1 CIIMAR/CIMAR-LA, Centro Interdisciplinar de Investigação Marinha e Ambiental, Universidade do Porto, Terminal de Cruzeiros do Porto de Leixões, Av. General Norton de Matos, S/N, 4450-208 Matosinhos, Portugal; ricardo.pereira@ciimar.up.pt (R.P.); anafbasto@gmail.com (A.B.); lvalente@icbas.up.pt (L.M.P.V.); 2 ICBAS, Instituto de Ciências Biomédicas Abel Salazar, Universidade do Porto, Rua de Jorge Viterbo Ferreira, 228, 4050-313 Porto, Portugal; 3 CBQF—Centro de Biotecnologia e Química Fina (Laboratório Associado), Escola Superior de Biotecnologia, Universidade Católica Portuguesa, Rua Diogo Botelho, 1327, 4169-005 Porto, Portugal; mpintado@ucp.pt

**Keywords:** aquaculture, sustainability, functional aquafeeds, oxidative stress, fruit peels, vitamin E, phenolic compounds, European sea bass

## Abstract

This study investigates the effects of pineapple by-products on feed preservation during storage at two different temperatures (25 °C and 4 °C) and on European sea bass (*Dicentrarchus labrax*) stress resistance. Four isoproteic, isolipidic, and isoenergetic diets were manufactured: CTRL—negative control, commercial diet without added antioxidants; VITE—positive control, CTRL diet with 100 mg kg^−1^ of vitamin E; and P2 and S2—VITE diet with 2% pineapple peel or stem flour, respectively. The fish (13.5 ± 0.8 g) were split into four replicate groups per diet and fed ad libitum for 12 weeks, after which they were subjected to a stress challenge of air exposure (1 min) followed by confinement (5 min, 100 kg m^−3^). Despite storage time lowering the antioxidant properties of all diets, P2 and S2 showed increased antioxidant capacity (DPPH^•^, ABTS^•+^, and ORAC) before and after storage. The diets were well accepted by the fish, and the VITE-fed fish showed significantly lower lipid peroxidation values in the liver and muscle compared to all remaining diets. However, pineapple by-product inclusion did not result in increased fish stress resistance. Further optimization is required for the successful use of pineapple by-products as natural antioxidants in aquafeeds.

## 1. Introduction

Aquaculture practices have grown exponentially over the last two decades, while simultaneously becoming more integrated into global food systems [1]. In 2020, this sector supplied 214 million tons of aquatic food for human consumption, including 88 million tons of fish, accounting for 56% of worldwide fish consumption [1,2]. The rapid growth of the sector has been primarily driven by the depletion of wild fish stocks and the ever-increasing demand for fish from a growing global population [3,4]. This underscores the need for continuous improvement and optimization of the sector in order to ensure a sustainable value chain.

Among the many research topics aimed at improving both the output quality and quantity of aquaculture production, the development of novel aquafeeds that promote fish welfare within a sustainability context is of particular interest [5]. A significant aspect of feed modulation efforts focuses on the inclusion of antioxidants to avoid feed oxidation and fortify fish antioxidant defenses [2]. Essentially, the aquafeed manufacturing process usually requires adding oil to provide feed with monounsaturated fatty acids (MUFAs) and polyunsaturated fatty acids (PUFAs) [6]. However, these fatty acids are highly susceptible to oxidation through free radical chain reactions initiated by peroxides and hydroperoxides [6]. This oxidation process can be ameliorated by antioxidant inclusion, subsequently avoiding feed staling and rancidity [7]. Moreover, farmed fish are commonly subjected to stress factors, mainly due to periodic handling and transportation [8]. In this context, antioxidants play a crucial role in maintaining fish cell homeostasis and preventing the intracellular formation of reactive oxygen species (ROS), which can lead to oxidative stress and, ultimately, lipid and protein oxidation, DNA damage, enzymatic inactivation, precocious cell aging, and even apoptosis [9,10,11,12].

Simultaneously, there is a growing trend among consumers towards natural antioxidants, as opposed to synthetic sources [13]. The most commonly used synthetic antioxidants in aquafeeds, such as ethoxyquin (6-ethoxy-2,2,4-trimethyl-1,2-dihydroquinoline, E324), BHT (2,6-diterc-butil-*p*-creso, E321), and BHA (2,3-terc-butil-4-hidroxianisol, E320), raise concerns regarding bioaccumulation, immune system inhibition, genotoxicity, endocrine disruption, carcinogenic potential, and tumor-promoting effects [14,15,16]. In 2022, the European Union (EU) definitely prohibited the use of ethoxyquin as an antioxidant feed additive (EU 2022/1375, EU2022) [17]. Therefore, the feed industry is under external pressure to find safe and natural alternative compounds with strong antioxidant activity [5,13,18,19,20,21].

In recent years, the global annual production of fruits and vegetables has doubled from 30 to 60 million tons [22]. This generates a steady output of antioxidant-rich by-products, such as fruit peels and stems, which are often discarded after processing [23]. This practice not only results in increased waste but also has adverse environmental consequences [24,25]. Specifically, fruit by-products are rich in vitamins, carotenoids, and polyphenols, all of which have been proven to be good sources of antioxidants [26,27].

Indeed, beyond simply acting as radical scavengers, the antioxidant compounds in fruit peels may also increase the baseline activity of antioxidant enzymes, fortifying oxidative defense mechanisms [26,28]. As a result, lipid peroxidation effectively decreases, preserving the nutritional value of fish flesh intended for human consumption [29,30]. In addition, when added to aquafeeds as exogenous antioxidant sources, fruit peels play a central role in modulating the fish immune system, leading to improved disease resistance and heightened resistance to xenobiotics [29,30,31,32]. Thus, these bioactive compounds can be used to enhance the antioxidant capacity in aquaculture feeds, as well as to boost the immunological and antioxidant defense system of farmed fish, which may even lead to improved fish robustness [30,31]. However, the modulation of fish antioxidant defenses through natural antioxidants depends on the fish species, with factors like the antioxidant source, the feed matrix, and the method and percentage of inclusion influencing the response [26]. Additionally, physico-chemical elements linked to feed manufacturing processes such as extrusion, pelleting, drying, and conditioning—namely varying levels of exposure to light, water and heat, pressure, and other forms of mechanical stress—can all affect the stability and effects of natural antioxidants [33]. However, the literature concerning the effects of natural antioxidant inclusion on the antioxidant capacity of aquafeeds, as well as its subsequent effect on fish antioxidant response, remains scarce [34]. Specifically, pineapple is a tropical fruit known for its sweet and tangy flavor that offers several health benefits, including antioxidant properties, which are highly modulated by its richness in various antioxidant polyphenolic compounds, including flavonoids and phenolic acids [35]. Despite its phenolic content being potentially lower than that of other widely commercialized fruits like berries [36], pineapple stands out due to its significant surplus of antioxidant-rich by-products within the context of the food industry, such as peels and stems [37], which are mostly discarded for composting [38], thus facilitating their inclusion in aquafeeds in terms of availability and market price. Moreover, the available literature highlights the potential of pineapple waste as a cheap, sustainable alternative feed additive with myriad benefits [34]. Indeed, the inclusion of pineapple peel powder in aquafeeds has proven to be a viable source of bioactive components, showing potential for increasing immune response, related gene expression, as well as growth efficiency in Nile tilapia (*Oreochromis niloticus*) [39]. Additionally, a study conducted by our research group demonstrated that incorporating 2% pineapple or mango peel flour in aquafeeds can increase the antioxidant content and capacity of aquafeeds compared to a control diet under the same manufacturing conditions [27].

This study hypothesizes that incorporating pineapple by-products in aquafeeds, alongside a minimal dose of vitamin E (100 mg kg^−1^), can enhance both feed and fish antioxidant capacity. Thus, the main objective was to evaluate their potential as natural antioxidants in diets for European sea bass (*Dicentrarchus labrax*) juveniles. Specifically, this study aimed to (a) assess the modulating effects of 2% pineapple peel flour or stem flour in fish antioxidant defenses, immune response, and overall stress response and (b) study the impact of these by-products on the preservation of the antioxidant capacity of the feed at different storage conditions.

## 2. Materials and Methods

### 2.1. Flours and Experimental Diets

Pineapple (*Ananas cosmosus* L.) peel and stem flours were obtained from AgroGrIN Tech^®^ (Porto, Portugal), a Portuguese start-up company specializing in the sustainable valorization of fruit and its by-products. To manufacture both flours, pineapple peels and pineapple stems were rinsed with tap water and homogenized in a JuiceMaster Professional liquid juicer (Mod. 42.8; Carico, Lausanne, Switzerland). The resulting homogenate’s solid component was collected, followed by a careful drying step in an oven at 60 °C for 48 h. The resulting dried biomass was then ground in a granite flour mill and sifted at 0.5 mm, yielding two distinct flours: pineapple peel flour and pineapple stem flour. Both products were kept in separate polyethylene bags and stored at −80 °C until further analysis and incorporation into the diets. The proximate composition and antioxidant capacity of the flours are described in Table 1. A commercial-based diet was formulated without adding any antioxidants. This diet was then extruded by SPAROS Lda. (Olhão, Portugal) and was used as a negative control (CTRL diet). Three experimental diets were then formulated by supplementing the CTRL dietary mix with a regular dose of 100 mg kg^−1^ vitamin E (Lutavit^®^ E50, BASF Nutrition, Ludwigshafen, Germany), creating a positive control (VITE diet), or by further adding 2% peel or stem flour to the VITE diet (P2 and S2 diets, respectively), substituting wheat meal. This was carried out during the feed mix preparation stage, before the extrusion process, and the inclusion percentage was decided based on an industry rationale that takes into account economic viability and data from previous studies [27].

### 2.2. Feed Trial and Sampling

For the feeding trial, 240 European sea bass juveniles were acquired from the commercial fish farm Aquicultura Balear S.A.U. (Culmarex Group, Murcia, Spain) and transported to the CIIMAR facility (Matosinhos, Portugal). After 2 weeks in quarantine, the fish were anesthetized with 2-phenoxy-1-etanol (200 µL L^−1^), and individually weighed (g) and measured (total length, cm). Homogeneous groups of 17 fish (13.5 ± 0.1 g of initial weight; 11.1 ± 0.1 cm of initial total length) were randomly distributed into 16 square fiber-glass tanks (50 L per tank, 4.6 kg m^−3^), establishing 4 replicate groups for each. The fish were fed thrice a day (8h30, 12h00, and 16h00) until apparent satiation using automatic feeders for 81 days. Manual feeding was performed after the automatic feeders to ensure that the fish were fed to satiety, and quantities dispensed by the automatic feeders were proportionally adjusted on a daily basis. The water temperature was maintained at 22.0 ± 1.0 °C, salinity at 35.0 ± 0.5‰, and water oxygen levels at a minimum of 90% saturation. Redox potential (300 mV), pH levels (7.5), and salinity were recorded daily. Total ammonium, nitrite, and nitrate were monitored twice a week and maintained at levels ≤0.05 mg L^−1^, ≤0.5 mg L^−1^, and ≤50 mg L^−1^, respectively, as is recommended for marine fish species [40]. After a 24 h fasting period, 10 fish from the initial stock and 20 fish per treatment by the end of the growth trial were sacrificed by an anesthetic overdose (2-phenoxy-1-ethanol, 500 µL L^−1^) and stored at −80 °C for whole-body composition analysis. After the growth trial and a 24 h fasting period, 24 fish per treatment were immediately sampled, while another subset of 24 fish per treatment was further subjected to a stress challenge involving air exposure for 1 min and confinement at 100 kg m^−3^ for 5 min, replicating common aquaculture practices. After the stress challenge, the fish were returned to their original tanks with the same density as the growth trial (4.6 kg m^−3^) and were allowed a 2 h recovery period prior to sampling. All fish were slightly anesthetized with 2-phenoxyethanol (200 µL L^−1^) for blood collection from the caudal vein using heparinized syringes, and centrifuged at 10,000× *g* for 5 min at 4 °C. The collected plasma was stored at −80 °C until analysis. The fish were then sacrificed with a sharp blow to the head, after which the intestine and liver were collected and weighed for the calculation of somatic indexes. Liver and left dorsal muscle samples from each fish were immediately frozen in liquid nitrogen and kept at −80 °C until further analysis. All sampled fish were individually weighed (g) and measured (total length, cm).

### 2.3. Chemical Analysis

Freeze-dried whole fish, flours, and diets were ground and homogenized prior to proximate composition analysis. All samples were analyzed in duplicates for ash, dry matter (DM), crude protein (N × 6.25), lipids, and gross energy, according to AOAC methods [41] as described by Basto et al. [42].

### 2.4. Radical-Scavenging Capacity and Lipid Peroxidation in Flours, Experimental Diets, and Fish Muscle

The antioxidant capacity of the selected flours was analyzed only before the manufacturing process, while the experimental diets were analyzed at two different time points: immediately after the manufacturing process and following an 81-day period, corresponding to the duration of the growth trial. During this period, the diets were stored at two different temperatures, 4 °C and 24 °C, to assess the impact of storage conditions on their antioxidant content and capacity. The diets were mixed with methanol:water (80:20 v/v) in a proportion of 1:10, blended using an IKA T 25 digital ULTRA-TURRAX^®^ (IKA, Staufen im Breisgau, Germany), and centrifuged at 5000× *g*. The supernatant, i.e., the free phenolic compound extract, was collected and stored at −80 °C until further analysis, while the remaining pellet was used for bound phenolic compound extraction. The pellet was hydrolyzed with 4 M NaOH in distilled water at room temperature using an orbital shaker (250× *g* for 3 h). The mixture was acidified to pH 1.5–2.0 via the addition of 32% HCl. Then, the acid mixture was washed three times with ethyl acetate. The resulting fraction was collected and totally dried using a rotatory vacuum evaporator at 30 °C. The resulting residues were then dissolved in pure methanol, generating bound phenolic extracts. Quantification of phenolic compounds was performed using the Folin–Cocialteu method, as described by Ainsworth and Gillespie [43]. Radical scavenging activity was measured using three assays: the ABTS^•+^ assay, performed using the method described by Sánchez-Moreno [44] and adapted by Gonçalves et al. [45]; the DPPH^•^ assay, performed according to the method of Brand-Williams et al. [46] and adapted by Gómez-García et al. [47]; and the ORAC assay, performed according to Apak et al. [48] as applied by Gómez-García et al. [47]. These analyses were conducted for both the free and bound extracts of the diets using a multi-detection plate reader (Synergy H1 HU126, Winooski, VT, USA). Calibration curves were generated using Trolox standards. The results were expressed as nmol of gallic acid equivalents (GAE) per mg of diet for the Folin–Cocialteu method, or as Trolox equivalents per mg of diet for all remaining analyses.

Fish muscle samples from both the non-stressed and stressed groups were processed for analysis according to Valente et al. [49]. Briefly, the fish muscle samples were subjected to acid hydrolysis using a glycine-HCl 32% buffer (0.1 M). Pepsin solution was then added, and the samples were incubated for 6 h at 37 °C in an oscillator at 120 rpm. After this, the samples were heated for 5 min at 100 °C to inactivate the enzyme and centrifuged, following the addition of 1 M NaOH to neutralize the pH value. ABTS^•+^ and ORAC radical scavenging potential was measured using the methodologies described for diets by Valente et al. [49] and Ribeiro et al. [50], respectively. Calibration curves were developed using Trolox standards. The results were expressed as nmol of Trolox equivalents per mg of fresh tissue for all remaining analyses. For lipid peroxidation evaluation, the muscle samples were homogenized using phosphate buffer (0.1 M, pH 7.4) in a proportion of 1:10 (*w*:*v*). Lipid peroxidation (LPO) was assessed using the methods described by Bird and Draper [51] through the quantification of thiobarbituric acid reactive substances (TBARSs), which are mainly composed of malondialdehyde (MDA), a by-product of the decomposition of fatty acids (PUFAs) induced by chemically unstable hydroperoxides. Absorbance was measured at 535 nm at 25 °C, and the rate of LPO was expressed as nmol of TBARS formed per g of muscle. All analyses were performed using a multi-detection plate reader (Synergy H1 HU126, Winooski, VT, USA).

### 2.5. Oxidative Stress Biomarkers in Fish Liver

Fish livers from both the non-stressed and stressed groups were homogenized using phosphate buffer (0.1M, pH 7.4) in a ratio of 1:10 (w v^−1^). To 300 µL of homogenate, 5 µL of butylated hydroxytoluene (BHT, 4%, diluted in methanol) was added. Thus, aliquots for LPO and carbonyl compounds (CCs) were made and subsequently stored at −80 °C. The remaining homogenate was centrifuged (10,000× *g*, 4 °C, 20 min), after which post-mitochondrial supernatant (PMS) was extracted and stored at −80 °C. The protein content of PMS was measured according to Bradford [52] in order to standardize the antioxidant enzyme activity measurements.

Catalase (CAT) activity was recorded in accordance with Greenwald [53], using hydrogen peroxide (H_2_O_2_, 30%) as the substrate. Absorbance was registered at 240 nm at 25 °C, and the results were expressed in µmol of H_2_O_2_ consumed per minute per mg of protein. Glutathione peroxidase (GPx) activity was assessed as reported by Mohandas et al. [54], also using H_2_O_2_ as the substrate and including sodium azide (NaN_3_) to inhibit CAT. Absorbance was measured at 340 nm at 25 °C, and the results were expressed as nmol of oxidized nicotinamide adenine dinucleotide phosphate (NADPH) per minute per mg of protein. Following the methods described by Habig [55], glutathione s-transferase (GST) was determined via the measurement of the conjugation between 1-chloro-2,4-dinitrobenzene (CDNB) and reduced glutathione (GSH). Absorbance was recorded at 340 nm at 25 °C for 5 min, and enzyme activity was expressed in nmol of CDNB conjugate formed per minute per mg of protein. The activity of glutathione reductase (GR) was assessed according to Cribb et al. [56] via consumption of NADPH measured at 340 nm for 3 min at 25 °C. The results were expressed in nmol of oxidized NADPH per minute per mg of protein. Total glutathione (TG) and oxidized glutathione (GSSG) were measured at 412 nm via the formation of 5-thio-2-nitrobenzoic acid (TNB), in accordance with Baker et al. [57]. The results were expressed as nmol of conjugated TNB formed per min per mg of protein. LPO was measured using the methodologies described for muscle, and the results were expressed as nmol of TBARS formed per g of liver. The total antioxidant capacity of fish liver was measured using a total antioxidant capacity assay kit (Sigma MAK187, Saint Louis, MO, USA), according to the manufacturer’s instructions, and the results were expressed in nmol per mg of tissue.

### 2.6. Acute Stress Response, Energetic Metabolism, and Immune System Status in Fish Plasma

Glucose, lactate, triglycerides, cholesterol, non-esterified fatty acids (NEFAs), and cortisol in fish plasma were assessed using commercial kits (1001190, 1001330, 1001313, 1001090, Spinreact, Girona, Spain; 434-91795 NEFA-HR (2) R1 and 436-91995 NEFA-HR (2) R2, Wako Chemicals, Neuss, Germany; RE52611, Tecan, Männedorf, Switzerland, respectively), following the manufacturer’s instructions adapted to a microplate format. For immune system status analysis, peroxidase activity was measured using the procedure described by Quade and Roth [58] and adapted by Costas et al. [59]. Lysozyme activity was obtained following the protocol developed by Parry et al. [60], with adaptation for a microplate by Hutchinson and Manning [61]. Complement activity (ACH50) was analyzed using the protocol described by Sunyer and Tort [62], using rabbit blood.

### 2.7. Statistical Analysis

Data were tested for normality and homogeneity of variances, considering the Kolmogorov–Smirnov and Levene’s tests, respectively, and, if necessary, appropriately transformed. Both one- and two-way ANOVA were used to analyze data, considering diet as a fixed factor for the one-way ANOVA, and diet and stress as fixed factors for the two-way ANOVA. The analyses were performed using Statistica v13.5 (TIBCO Software Inc., Palo Alto, CA, USA) software. When significant effects were found, a pairwise multiple comparison test (Tukey HSD) was carried out.

## 3. Results

### 3.1. Chemical Composition and Antioxidant Capacity of Pineapple Flours and Diets Before and After Storage

The proximate composition and antioxidant content of both the peel and stem flours are depicted in Table 1. Both flours presented very similar protein and fat content, ash, and gross energy values. The pineapple peel flour showed a higher total quantification of phenolic compounds, as well as higher DPPH^•^ radical scavenging capacity and ORAC in the extracts of free phenolic compounds compared to the stem flour. The same trend was observed for bound phenolic extracts. Besides, pineapple peel flour also showed higher ABTS^•+^ radical scavenging capacity for bound phenolic compounds. Lipid peroxidation values were higher in pineapple peel four than in pineapple stem flour.

Proximate composition of the diets is described in Table 2. The 2% inclusion of these flours in the experimental mix did not affect the diets’ proximate composition. Moreover, immediately after extrusion (initial sampling point), both the total free and bound phenolic compounds were highest in diet P2 (Table 3).

Diet antioxidant capacity is presented in Table 3. VITE and P2 diets showed the highest DPPH^•^ and ABTS^•+^ radical scavenging capacity, as well as the highest ORAC values in free phenolic extracts. But in the bound phenolics, the higher antioxidant capacity was evidenced in diet S2. Overall, and regardless of temperature, storage time was found to modulate free phenolic compound concentrations, which decreased in the VITE diet. However, during storage, diets with pineapple by-product flour (P2 and S2) maintained their free phenolic compounds.

Moreover, antioxidant potential was also modulated in both free and bound phenolic extracts. Specifically, the DPPH^•^ radical scavenging capacity of the bound extracts from diets stored at 24 °C decreased in S2, while increasing in VITE and P2. The ABTS^•+^ radical scavenging capacity of the free phenolic extracts was lower in P2 and S2, which in turn increased the ABTS^•+^ radical scavenging capacity of the bound phenolic extracts. In the free phenolic extracts, VITE, P2, and S2 were associated with increased ORAC after storage, while in the bound phenolic extracts, all diets presented increased ORAC after storage. Finally, although lipid peroxidation increased after storage in all diets, levels were similar between storage at 4 or 24 °C. However, post-storage values for diets with added synthetic vitamin E, regardless of pineapple flour inclusion, were overall lower than those from the CTRL diet. In terms of storage temperature, the diets were mostly equally affected by different storage temperatures, with a few exceptions. The ABTS^•+^ radical scavenging capacity of free phenolic extracts of the CTRL increased at 24 °C storage. In sum, the addition of these flours induced an increase in the DPPH^•^ radical scavenging capacity of bound phenolic extracts in P2 compared to the remaining diets, while the ABTS^•+^ radical scavenging capacity of the diets across all sampling times increased in the free phenolic extracts of S2, followed by P2, compared to CTRL and VITE. Moreover, the ORAC of free and bound phenolic extracts is higher in P2 after storage.

### 3.2. Growth, Intake, Whole-Body Composition, and Somatic Indexes

As shown in Table 4, all diets were well accepted by the fish, which almost quintupled in weight after 12 weeks. There were no significant differences in growth performance, feed intake, whole-body composition, or somatic index.

### 3.3. Oxidative Stress Biomarkers in the Liver and Muscle

**Figure 1:**
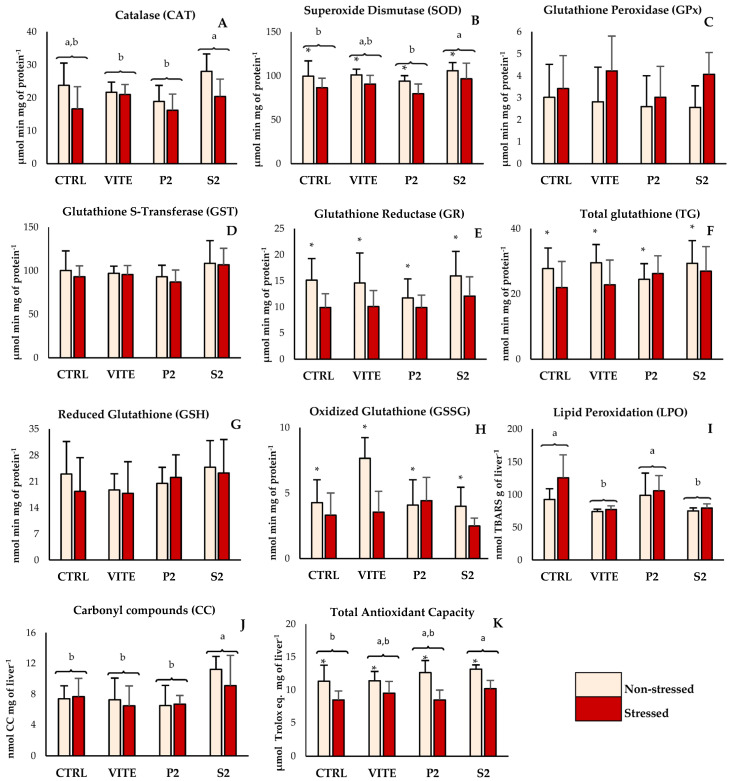
Antioxidant enzyme activity, glutathione quantification, lipid and protein oxidation, and total non-enzymatic antioxidant capacity of liver from fish fed with the experimental diets. Values are presented as mean ± standard deviation (n = 12). A 2-way ANOVA was performed, where different superscript lowercase letters (a,b) indicate differences between diets, while “*” indicates differences between the stressed and non-stressed groups (*p* < 0.05).

**Figure 2:**
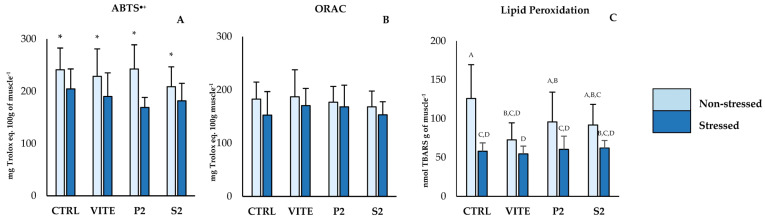
Lipid peroxidation values in fish muscle. Values are presented as mean ± standard deviation (n = 12). A 2-way ANOVA was performed, where “*” indicates differences between the stressed and non-stressed groups, and different superscript uppercase letters (“A, B, C, D”) indicate interaction differences between the variables “Stress” and “Diet” (*p* < 0.05).

The inclusion of pineapple by-products into the diet resulted in a significant alteration in oxidative biomarkers in fish liver (Figure 1A,B,F,I–K). However, applied stress affected the parameters associated with antioxidant defenses and oxidative stress in Figure 1B,E,F,H,K, resulting in a downregulation of their activity. Specifically, catalase (CAT) activity displayed significant differences between diets (Figure 1A); fish fed with the S2 diet had higher CAT activity compared to the positive control (VITE) and to the P2 diet group, regardless of their stress condition. Superoxide dismutase (SOD) activity showed differences between diets, as well as between the stressed and non-stressed groups. Fish fed S2 had higher SOD activity compared to the CTRL and to P2, despite not differing from the VITE group. The non-stressed groups showed significantly higher values of SOD activity for all diets compared to the corresponding stressed groups. GR activity and TG content did not differ between diets, but the non-stressed group displayed higher values compared to the stressed groups. The results obtained for GSSG reflected these differences, as the activity of this enzyme was also significantly higher in the non-stressed groups compared to the stressed groups. However, the LPO data showed that VITE and S2 had significantly less TBARS formation compared to CTRL and P2. Protein oxidation differed between diets, with fish fed S2 showing significantly higher CC concentrations than all other diets (Figure 1J). And finally, total antioxidant capacity (TAC) showed significant differences both between diets and between stressed and non-stressed groups. While the non-stressed group showed higher values than the stressed group, fish fed with S2 were the only group that showed significantly higher TAC compared to the negative control (CTRL). In muscle tissue (Figure 2A–C), both ABTS^•+^ and LPO values were reduced in the stressed group. However, there were no significant differences between diets within the same stress group.

### 3.4. Acute Stress Response, Energetic Metabolism, and Immune System Status in Fish Plasma

Levels of lactate, glucose, and cortisol were shown to be significantly higher in the stressed group, but no differences could be observed between diets (Table 5). A significant interaction was observed among the “diet” and “stress” factors concerning the NEFA levels. In the non-stressed group, fish fed with the VITE diet had significantly lower levels of NEFA compared to those fed with P2 and S2. However, no significant differences could be perceived between diets in the stressed group. Moreover, in terms of innate immune system parameters, no differences were observed between the groups fed with the different diets (Table 5). But after stress, ACH50 values decreased in all fish, while peroxidase values increased.

## 4. Discussion

Antioxidants play a key role in preventing feed staling and rancidity, which in turn preserves the nutritional value of the feed while simultaneously reducing costs associated with feed waste, handling, and storage [7,63].

Economic viability is essential for implementing alternative sources in aquaculture, but data on the retail pricing of pineapple peels and stems are lacking, as these by-products are not typically commercialized [64]. In turn, this could lead to low market value if demand were to be created within a circular economy framework [65]. Moreover, processing expenses are not included in this estimation, emphasizing the need for an economically viable retail price for these by-products. Thus, an industrial rationale was adopted in this work, choosing a conservative inclusion level of 2% to ensure practicality and economic feasibility in industrial settings, considering both efficacy and cost-effectiveness. This study has shown that adding 2% pineapple by-product flours elevated the levels of both free and bound phenolic compounds, but this was not reflected in fish antioxidant activity as a physiological response towards stress conditions.

The role of phenolic compounds as primary antioxidants is well established [66,67,68,69], and pineapple is known to be rich in these compounds [70]. Overall, the results from the present study confirm this, given that both types of pineapple waste flours, namely peel and stem, were relatively rich in terms of quantity and variety of phenolic compounds. Nevertheless, pineapple peel flour presented higher antioxidant properties, along with a higher total content of free and fiber-bound phenolic compounds compared to pineapple stem flour. Indeed, these results align with previous studies reporting that pineapple peel has higher levels of phenolic content and greater radical scavenging activity compared to pineapple stem [71]. In addition, Huang et al. [71] also reported that pineapple peel is richer in crude fiber than pineapple stem, which could partially explain the higher amounts of fiber-bound phenolic compounds found in pineapple peel in the present study. Indeed, pineapple is rich in fiber-bound phenolic compounds, mainly flavonoids and phenolic acids [35,70]. In this study, while the final amounts of fiber-bound phenolic compounds did not vary greatly between all diets, it is known that one of the main influencers of antioxidant activity is the antioxidant compound composition, as certain antioxidants are more potent than others, which subsequently applies to different phenolic compounds [72]. In the present study, the inclusion of pineapple by-product flours (diets P2 and S2) leads to a higher extraction yield of free phenolic compounds compared to VITE and CTRL. Moreover, while bound phenolic compound extraction yield was similar between diets, feed radical-scavenging properties associated with bound phenolic compound extracts were higher in P2 and S2 compared to the VITE positive control, even after storage. This is in concordance with a previous study by our research team [27], which showed that pineapple peel flour fiber is rich in transferulic and 4-coumaric acids, both of which are potent antioxidants that exhibit considerable radical-scavenging activity [73,74], and can in fact increase aquafeed radical scavenging capacity in terms of DPPH^•^, ABTS^•+^ and ORAC. Indeed, available information shows that bound phenolic compounds are more resistant to oxidation than free forms, mainly due to the action of abiotic factors, including interactions with dietary fiber, altering their bioaccessibility and bioactivity [75]. Moreover, the antioxidant potential of by-product matrices like fruit peels is determined not only by their overall phenolic content, but also by the particular profile of the phenolic compounds present [76], their specific chemical structure, and the subsequent interactions with various macromolecules, such as fiber, protein, lipids, and carbohydrates [77].

Low-moisture food matrices have been demonstrated to be less prone to oxidation over time and less affected by other abiotic factors such as temperature [78]. However, this study revealed a notable increase in lipid oxidation after storage, indicating that storage time still seems to be a pro-oxidant factor that clearly affects the antioxidant properties of aquafeeds. Nevertheless, the present results reinforce that feed antioxidant properties were improved with a concomitant inclusion of vitamin E and a 2% inclusion of pineapple by-products flour, mostly due to increases in the antioxidant capacity of DPPH^•^ of free phenolic extracts of diets stored at 4 °C, as well as in ABTS^•+^ and ORAC antioxidant capacity of fiber-bound phenolic extracts at both storage times. While it is widely acknowledged that temperature plays a pivotal role in lipid stability, surprisingly, refrigerated storage at 4 °C did not alter lipid peroxidation compared to storage at 24 °C, highlighting the fundamental role of other abiotic factors in the lipid peroxidation process in low-moisture food matrices. Overall, refrigerated storage of feeds might be undesirable from an economic standpoint, as it can impose an economic burden that might not be sustainable. It is also important to note that in this study, the variables of air exposure and light exposure were eliminated, both of which are deciding factors in oxidation reactions that could possibly be catalyzed by temperature [79]. Nonetheless, data from this work did not show evidence of increased lipid peroxidation in diets after storage at both temperatures, meaning that further research is required in order to lessen the antioxidant potency of these diets during storage. However, while experiments with lower temperatures such as −20 °C or −80 °C could present improvements in the antioxidant capacity preservation of these feeds, as these temperatures are better for maintaining the biochemical structure of natural antioxidants [80], additional studies on the economic viability of these storage temperatures is required. Overall, adjusting by-product inclusion percentages and diet manufacturing methods may improve feed antioxidant properties and, consequently, fish stress resistance.

Literature concerning the inclusion of fruit by-products in aquafeeds is scarce and shows controversial results regarding their impact on fish growth. These differences hinge on various factors such as species, the specific fruit by-product used, and the method and percentage of inclusion [29,30,31,32,81]. In the present study, experimental diets did not significantly affect fish growth performance, feed intake, or whole-body composition, demonstrating that a 2% inclusion of pineapple flours in aquafeeds for European sea bass was well accepted by the fish. The supplementation of European sea bass diets with 100 mg kg^−1^ of vitamin E led to no differences in terms of exogenous antioxidant response, as none of the analyzed enzymes or glutathione in the VITE-fed fish showed significant differences compared to the CTRL group. However, fish fed with VITE exhibited significantly less lipid peroxidation values in both liver and muscle compared to CTRL, meaning that the added vitamin E acted as an exogenous antioxidant source to lessen lipid peroxidation, thereby obviating the necessity to produce more antioxidant enzymes and/or glutathione [82]. Indeed, extensive research has demonstrated that α-tocopherol exhibits remarkable efficacy in scavenging ROS [83], since by reacting with peroxyl radicals (LOO^•^), α-tocopherol effectively disrupts the chain reactions that lead to lipid and protein peroxidation [84], ultimately safeguarding against the oxidation of proteins and lipids. Consequently, synthetic vitamin E is widely used as an antioxidant in aquaculture, particularly as an alternative to ethoxyquin, which was banned by the European Union [17]. Recommended vitamin E doses in fish dietary formulations vary between 25 and 200 mg kg^−1^, contingent upon the species and maturation state [12]. Despite the demonstrated importance of vitamin E supplementation in the development of European sea bass larvae [85], limited data exist regarding its antiperoxidative effects in commercially sized fish. Gatta et al. [86] proposed that diets for European sea bass (IBW = 208 g) should contain α-tocopherol levels ranging from 254 mg kg^−1^ to 942 mg kg^−1^ to effectively mitigate lipid oxidation. However, previous work from our research team showed no differences in market-sized European sea bass antioxidant systems when fed with either 100 or 500 mg kg^−1^ [87]. This evidence served as a basis for the vitamin E inclusion percentage used in the present study. Indeed, based on the rich antioxidant profile and quantities present in pineapple, the concomitant inclusion of 2% pineapple by-product flour and 100 mg kg^−1^ were included in this work with the purpose of offering additional antioxidant and immune system protection to farmed fish beyond the standard 100 mg kg^−1^ vitamin E supplementation. This allows for a cheap, and therefore most realistic, method of inclusion for an aquaculture scenario within the context of a circular economy, as more expensive methods of inclusion (i.e., liquid extracts, bioencapsulation, etc.) would greatly increase costs and thus render the experiment unrealistic in an industrial setting.

Indeed, when antioxidants are included in animal diets, many external factors impact their antioxidant effectiveness and bioavailability within the organism [88]. Subsequently, this impacts their capacity not only to act as exogenous antioxidant sources, but also to upregulate the innate antioxidant system defenses of farmed fish, i.e., production of antioxidant enzymes [88]. Specifically, enzymes such as CAT and SOD play a crucial role in converting harmful free radical molecules into non-damaging substances [82]. Thus, the increased production of CAT and SOD observed in the livers of fish fed with the pineapple stem diet (S2) suggests that S2 might induce additional intracellular oxidative stress, necessitating further production of antiperoxidative enzymes to maintain lipid oxidation levels. Additionally, the concentration of carbonyl compounds was higher in the S2 fish, indicating that increased CAT and SOD activity was insufficient to prevent the oxidative damage of proteins promoted by ROS [82].

In this work, the activity of SOD, GR, TG, and GSSG exhibited distinct changes following the stress challenge, confirming the fish’s physiological response to acute stress. Additionally, the liver’s TAC was also impacted by stress, suggesting that intracellular molecules possessing antioxidant properties were likely oxidized due to the increased formation of ROS [82]. However, no differences in protein oxidation and lipid peroxidation were observed between the non-stressed and stressed groups, further indicating that the natural antioxidants present in these by-products were not potent enough to provide additional antioxidant protection to the fish compared to those fed the positive control diet (VITE) [84]. In terms of differences in the “Diet” variable, the liver activity of SOD in fish fed the S2 diet was higher compared to those on the CTRL diet, while lipid peroxidation was lower in the S2 group. This is in concordance with the findings from other studies, indicating the increased requirement for higher concentrations of enzymes to counter lipid peroxidation [89]. However, the P2 diet yielded results similar to VITE in terms of CAT activity, suggesting that it did not provide significant benefits to the fish. GR enables the regeneration of GSH from GSSG, thereby aiding in the regulation of oxidative stress [82]. The results demonstrate that differences in GSSG between the non-stressed and stressed groups affected the overall levels of TG. This decrease in GSSG in the stressed group seems to be responsible for the decrease in TG in fish liver. Since GSH is a necessary cofactor for many antioxidant enzymes such as GPx and GST, and also has direct antioxidant and ROS-chelation properties [90], the surge of intracellular ROS likely necessitated a higher production of GSH through the reduction of GSSG mediated by GR [82]. Non-enzymatic TAC exhibited differences between diets, with the S2-fed fish displaying significantly higher TAC than the CTRL fish. This tendency suggests that fish fed with the S2 diet may, to some extent, exhibit enhanced non-enzymatic antioxidant capacity in the liver. Despite this, findings from this study reveal that fish fed with different diets exhibited variations in LPO and protein oxidation (CC). Specifically, none of the experimental diets showed significantly lower levels of LPO and CC than fish fed with the positive control (VITE), indicating that, ultimately, the addition of pineapple by-products did not provide additional antioxidant protection to fish liver. Meanwhile, in fish muscle, the non-stressed group displayed higher ABTS^•+^ radical scavenging capacity, with lipid peroxidation following a similar trend. But the overall values were lower, implying that the stressed group experienced a depletion of exogenous antioxidants in an attempt to reduce lipid peroxidation.

Overall, the inclusion of pineapple peel and stem flours in aquafeeds did not enhance fish liver antioxidant defenses or muscle antioxidant capacity. Indeed, given that the results show evidence of modulation of liver antioxidant activity in the P2 and S2 diets compared to VITE, the fact that no beneficial physiological effects were observed may be attributed to the low percentage of pineapple byproducts, including being unable to positively influence the antioxidant system of the fish. However, some negative effects were identified in fish fed with diets containing natural antioxidants from pineapple added to the VITE mix. The fact that the P2 group showed higher liver lipid oxidation than the VITE group, along with the fact that the S2 group showed higher rates of protein oxidation and required a higher production of CAT to achieve the same levels of lipid oxidation as VITE, implies that a higher inclusion percentage could also negatively impact the liver antioxidant system of fish fed with experimental diets containing natural antioxidants from pineapple by-products. In contrast, Guardiola et al. [91] reported favorable results in a trial with European sea bass using diets containing date palm extract, namely an increased antioxidant potential in plasma and an upregulation of genes associated with antioxidant enzymes in the gills and head kidney. This was achieved using a higher inclusion level (10%, 100 g kg^−1^ of feed) compared to the 2% inclusion used in this study. In another study using zebrafish (*Danio rerio*) fed diets containing mango peel (5–20%, 50–200 g kg^−1^ of feed), no differences were observed in terms of the activity of some antioxidant enzymes, as GPx and SOD remained unaffected [29]. Nonetheless, CAT activity rose at 200 g kg^−1^, while lipid peroxidation showed no differences at that inclusion level [29], possibly meaning that the organism required a higher CAT activity in order to maintain cell homeostasis compared to the control [82]. Moreover, in that same study, a higher level of whole-body lipid peroxidation was observed at 100 g kg^−1^ inclusion, while antioxidant enzyme activity remained unaffected [29]. In tambaqui (*Colossoma macropomum*) fingerlings, inclusion of lemon peel extract (0.025–2%, 0.25–2 g kg^−1^) improved the condition factor, the survival, and the activity of SOD in the liver [31], which is expected, since extracts are typically more concentrated than whole fruits in terms of antioxidant compound content [88,92], despite lacking economic viability in a realistic aquaculture scenario due to processing costs. Inversely, palm oil inclusion (1.5–6%, 15–60 g kg^−1^ of feed) in diets for African catfish (*Clarias gariepinus*) lowered lipid peroxidation in muscle [93]. However, this may be caused by the very high amounts of vitamin E present in palm oil, which is one of the most abundant natural sources of vitamin E, being rich in tocotrienols and tocopherols in amounts of up to 800 mg kg^−1^ [94].

When fish are faced with acute stress, cortisol stimulates glycogenolysis and gluconeogenesis, raising glucose levels to provide sufficient energy to cope with the metabolic demands resulting from stress [95]. In addition, during stress, increased oxygen demand can lead to cellular hypoxia, causing an increase in circulating lactate levels [96]. Indeed, the primary source of energy for fish to withstand unfavorable conditions is blood glucose, making it an efficient indicator of stress [81]. This response of increased cortisol, glucose, and lactate levels was verified in all treatments after 2 h of recovery from the induced stress challenge. However, the inclusion of 2% pineapple peel and stem flour had no impact on plasma stress biomarkers, evidencing no physiological benefit in an acute stress situation.

Regarding plasma metabolites, Di Marco et al. reported that elevated plasma NEFA levels are associated with the mobilization and subsequent oxidation of lipid reserves due to increased metabolic demand [97]. Moreover, this rise in NEFA levels, as suggested by Shearer et al. [98], might originate from sources like mesenteric fat or hepatic tissue, pointing toward the activation of transcription factors regulating metabolic pathways and nutrient transport. Such regulations potentially influence the plasma levels of NEFAs, triglycerides, and cholesterol. Although no differences were observed in terms of triglycerides and cholesterol, our results evidenced a slight tendency to decrease post-stress NEFA levels in fish fed with the P2 and S2 diets, whereas these levels increased in both control groups. This aforementioned tendency for decreasing NEFAs in the plasma of fish fed with P2 and S2 points towards the possibility that less NEFA is mobilized, and therefore less energy is required in order to ameliorate the negative effects of acute stress [97]. Indeed, this lipid-lowering tendency may be attributed to the effects of exogenous antioxidants, including phenolic compounds, which deter lipid peroxidation and the generation of lipo-peroxides, known contributors to atherosclerosis [81]. Actually, lipid-lowering activities in fish metabolism via the inclusion of natural antioxidant sources have also been reported via the inclusion of Doum palm fruit powder [81], clove basil leaf extract [99], and Aloe vera polysaccharides [100]. Further experimentation with higher inclusion percentages of pineapple by-products, or even different methods of inclusion within the context of economic viability, could possibly produce more favorable outcomes in terms of acute stress response. Finally, at the immunological level, significant differences were observed in the activity of ACH50 and total peroxidase activity between the stressed and non-stressed groups, as expected in response to stress [101]. However, the absence of significant differences between the diets indicates that the immune response was not reinforced by them.

## 5. Conclusions

This study demonstrates that incorporating 2% pineapple by-product flour (either from peels or stems) alongside a minimal dose of vitamin E (100 mg kg^−1^) enhances the antioxidant capacity of feed. Furthermore, the results indicate that feed can be stored at room temperature without refrigeration, effectively preventing oxidation and reducing associated costs.

While the results from the in vivo trial with European sea bass juveniles reinforce the importance of incorporating vitamin E into fish feeds, none of the diets containing natural antioxidants provided additional benefits in fish antioxidant defenses or overall stress resistance compared to the VITE diet. Further research is needed to optimize the production process of by-product flours to maximize antioxidant retention after processing. Adjusting the inclusion levels of these by-products and refining diet manufacturing methods may enhance the antioxidant properties of feed and, consequently, improve fish stress resistance. This optimization effort could involve experiments with different inclusion percentages of the by-product flours used in this study. However, economic viability must always be considered, as higher inclusion percentages may affect cost-effectiveness. Additionally, further optimization could include experimenting with alternative antioxidant sources and exploring various food matrices.

Such studies are crucial for achieving a successful upregulation of the antioxidant system of fish in order to improve their resistance to stress, subsequently improving their disease resistance and overall health, possibly leading to benefits in terms of production quantity and quality in the aquaculture sector in a sustainable manner within the context of a circular economy.

## Figures and Tables

**Table 1:** Proximate composition and antioxidant capacity of pineapple by-product flours.

	Peel Flour	Stem Flour
**Proximate composition ^1^**		
Dry matter	92.7 ± 0.1	89.4 ± 0.1
Crude protein	5.2 ± 0.01	4.8 ± 0.04
Crude fat	0.5 ± 0.01	0.9 ± 0.02
Ash	3.2 ± 0.01	2.8 ± 0.01
Gross energy	16.7 ± 0.2	17.8 ± 0.01
Carbohydrates	91.2 ± 0.1	91.4 ± 0.1
**Free phenolic extracts ^2^**		
DPPH^•^	322.0 ± 0.7	223.8 ± 2.6
ABTS^•+^	845.6 ± 10.3	941.0 ± 103.7
ORAC	3722.7 ± 65.9	1613.9 ± 74.9
Total phenolic compounds	1157.6 ± 97.8	622.6 ± 2.4
**Bound phenolic extracts ^2^**		
DPPH^•^	886.3 ± 143.6	702.7 ± 19.1
ABTS^•+^	9232.2 ± 931.6	6089.3 ± 101.7
ORAC	9377.5 ± 957.4	6487.2 ± 529.1
Total phenolic compounds	953.8 ± 13.2	502.1 ± 35.4
**Lipid peroxidation ^3^**		
TBARS	167.1 ± 1.5	121.0 ± 2.8

Values are presented as mean ± standard deviation (n = 3). ^1^ Proximate composition is expressed in % of dry matter (DM), except for gross energy, which is expressed in kJ g^−1^ DM. Carbohydrates are calculated by adding values from protein, ash, and lipids and subtracting that value from 100. ^2^ Antioxidant capacity of both extracts was measured via the DPPH^•^, ABTS^•+^, and ORAC radical scavenging capacities and is expressed in mg of Trolox equivalents (TE) per 100 g of DM. The total quantification of phenolic compounds in both extracts was measured using the Folin–Ciocalteu method and is expressed in mg of gallic acid equivalents (GAE) per 100 g of DM. ^3^ Lipid peroxidation is expressed in nmol TBARS g^−1^ of DM.

**Table 2:** Formulation and proximate composition of experimental diets.

	CTRL	VITE	P2	S2
**Ingredients (%)**				
Fishmeal Super Prime ^1^	15.0	15.0	15.0	15.0
Poultry meal ^2^	10.0	10.0	10.0	10.0
Porcine blood meal ^3^	2.0	2.0	2.0	2.0
Soy protein concentrate ^4^	15.0	15.0	15.0	15.0
Wheat gluten ^5^	6.0	6.0	6.0	6.0
Corn gluten meal ^6^	10.0	10.0	10.0	10.0
Soybean meal 44 ^7^	8.0	8.0	8.0	8.0
Sunflower meal 40 ^8^	5.0	5.0	5.0	5.0
Wheat meal ^9^	10.0	10.0	7.9	7.9
Wheat bran ^9^	4.1	4.1	4.1	4.1
Pineapple peel ^10^			2.0	
Pineapple stem ^10^				2.0
Vitamin and Mineral Premix PV02 ^11^	1.0	1.0	1.0	1.0
Vitamin C35 ^12^	0.03	0.03	0.03	0.03
Vitamin E50 ^12^		0.02	0.02	0.02
Yttrium (III) oxide	0.02	0.02	0.02	0.02
Monoammonium phosphate	0.97	0.97	0.97	0.97
Fish oil ^13^	7.0	7.0	7.0	7.0
Soybean oil ^14^	5.9	5.9	6.0	6.0
**Proximate composition ^15^**				
Dry matter	96.2 ± 0.3	98.1 ± 0.02	94.8 ± 0.02	97.1 ± 0.1
Ash	6.3 ± 0.05	6.4 ± 0.1	6.4 ± 0.03	6.4 ± 0.0
Protein	55.8 ± 0.02	55.1 ± 0.3	55.3 ± 0.1	56.3 ± 0.2
Lipids	17.3 ± 0.4	17.0 ± 0.4	16.7 ± 0.4	17.5 ± 0.5
Gross energy	22.5 ± 0.02	21.7 ± 0.1	22.7 ± 0.1	22.7 ± 0.01
Carbohydrates	20.4 ± 0.3	21.5 ± 0.3	21.3 ± 0.6	20.7 ± 0.4

Values are presented as mean ± standard deviation (n = 3). Ingredients are expressed in % of dry matter. ^1^ Peruvian Fishmeal Super Prime: 66.3% crude protein (CP), 11.5% crude fat (CF) (Pesquera Diamante, Lima, Peru). ^2^ Poultry meal: 62.4% CP, 12.5% CF (SAVINOR UTS, Trofa, Portugal). ^3^ Porcine blood meal: 89.1% CP, 0.4% CF (SONAC BV, Vuren, The Netherlands). ^4^ Soy protein concentrate: 62.2% CP, 0.7% (CF) (ADM, Rotterdam, The Netherlands). ^5^ Wheat gluten: 80.4% CP, 5.8% CF (Roquette, Lestrem, France). ^6^ Corn gluten meal: 61.2% CP, 5.2% CF (COPAM, Lisbon, Portugal). ^7^ Soybean meal 44: 43.8% CP, 3.5% CF (Ribeiro and Sousa Lda., Paredes, Portugal). ^8^ Sunflower meal (HiPro): 42.9% CP, 3.8% CF (AGP Slovakia, S.R.O., Komárno, Slovakia). ^9^ Wheat meal: 11.7% CP, 1.6% CF (Molisur, Alhaurín el Grande, Spain); Wheat bran: 15.2% CP, 4.7% CF (Ribeiro and Sousa Lda., Portugal). ^10^ Pineapple peel flour: 5.2% CP, 0.5 CF; pineapple stem flour: 4.8% CP, 0.5% CF (AgroGrIN Tech^®^, Portugal). ^11^ Vitamin and mineral premix: Vitamins are expressed in mg or IU per kg of diet: vitamin A (retinyl acetate), 20,000 IU; vitamin D3 (DL-cholecalciferol), 2000 IU; vitamin K3 (menadione sodium bisulfite), 25 mg; vitamin B1 (thiamine hydrochloride), 30 mg; vitamin B2 (riboflavin), 30 mg; vitamin B6 (pyridoxine hydrochloride), 20 mg; vitamin B12 (cyanocobalamin), 0.1 mg; vitamin B5 (pantothenic acid), 100 mg; vitamin B3 (nicotinic acid), 200 mg; vitamin B9 (folic acid), 15 mg; vitamin H (biotin), 3 mg; betaine, 500 mg; inositol, 500 mg; choline chloride, 1000 mg; vitamin C (stay C), 1000 mg. Minerals (% or mg/kg diet): manganese oxide, 9.6 mg; potassium iodide, 0.5 mg; cupric sulphate, 9 mg; cobalt sulphate, 0.65 mg; zinc oxide, 7.5 mg; sodium selenite, 0.01 mg; iron sulphate, 6 mg; sodium chloride, 2.41%; calcium carbonate, 18.6%; NaCl (sodium), 4%; WISIUM, Premix Lda., Viana do Castelo, Portugal. ^12^ Vitamins C (35%) and E (50%) (DSM Nutritional Products, Kaiseraugst, Switzerland). ^13^ Fish oil: 98.1% CF, of which 16% is eicosapentaenoic acid (EPA) and 12% is docosahexaenoic acid (DHA) (Sopropêche, Wimille, France). ^14^ Soybean oil: 98.6% CF (JC Coimbra, Coimbra, Portugal). ^15^ Proximate composition is expressed in % of dry matter (DM), with the exception of gross energy, which is expressed in kJ g^−1^ DM. Carbohydrates are calculated by adding values from protein, ash, and lipids and subtracting that value from 100.

**Table 3:** Antioxidant potential of experimental diets from before the growth trial and after storage at 4 °C and 25 °C for 12 weeks.

	CTRL			VITE			P2			S2		
	Initial	4 °C	24 °C	Initial	4 °C	24 °C	Initial	4 °C	24 °C	Initial	4 °C	24 °C
**Free phenolic extracts ^1^**												
DPPH^•^	187.0 ± 2.0	178.4 ± 25.6	229.4 ± 22.4	211.9 ± 6.8	185.4 ± 8.3	210.0 ± 25.3	194.2 ± 11.4	197.6 ± 2.3	180.1 ± 9.2	186.0 ± 4.5	195.1 ± 13.8	180.1 ± 14.5
ABTS^•+^	596.1 ± 4.9	577.9 ± 77.3	654. ± 8.9	689.0 ± 60.5	482.8 ± 14.0	563.9 ± 83.2	701.0 ± 5.0	518.8 ± 25.0	519.7 ± 22.6	597.1 ± 25.8	513.3 ± 33.5	518.4 ± 60.5
ORAC	1828.3 ± 176.6	2076.5 ± 73.7	1655.1 ± 278.3	2046.3 ± 8.0	2314.4 ± 202.7	2709.6 ± 39.1	1911.8 ± 52.1	2974.4 ± 213.8	2142.9 ± 64.2	1746.4 ± 161.9	2061.3 ± 120.5	1794.5 ± 229.3
Phenolic compounds	606.5 ± 11.2	585.4 ± 8.9	614.6 ± 15.4	646.0 ± 7.2	594.0 ± 8.5	592.7 ± 21.6	676.2 ± 11.7	652.3 ± 7.3	655.4 ± 29.3	645.3 ± 27.5	624.4 ± 11.3	631.8 ± 8.4
**Bound phenolic extracts ^1^**												
DPPH^•^	43.9 ± 10.6	50.8 ± 7.3	48.9 ± 4.1	35.4 ± 1.2	45.3 ± 2.8	55.7 ± 7.5	45.0 ± 5.2	51.4 ± 1.2	56.1 ± 1.8	62.3 ± 7.5	44.8 ± 2.6	42.1 ± 2.0
ABTS^•+^	71.3 ± 6.2	74.1 ± 3.0	62.9 ± 5.9	64.4 ± 4.8	70.4 ± 8.2	77.3 ± 8.5	76.1 ± 9.6	94.4 ± 8.1	87.8 ± 6.5	94.9 ± 2.2	114.9 ± 8.2	105.6 ± 7.1
ORAC	1315.4 ± 75.3	1750.1 ± 74.5	1399.6 ± 61.2	1086.7 ± 88.3	1708.7 ± 50.2	1477.3 ± 89.6	1306.5 ± 113.5	1994.0 ± 89.4	1926.2 ± 141.4	1329.7 ± 62.2	1567.1 ± 95.0	1507.7 ± 180.1
Phenolic compounds	265.3 ± 0.2	266.6 ± 4.3	257.9 ± 3.8	265.2 ± 1.0	269.9 ± 1.5	265.5 ± 4.5	279.5 ± 2.0	278.9 ± 0.4	282.0 ± 1.6	272.9 ± 0.8	272.7 ± 0.5	272.5 ± 0.7
**Lipid peroxidation ^2^**												
TBARS	59.7 ± 3.1	140.8 ± 4.3	140.6 ± 7.4	58.8 ± 3.5	133.6 ± 5.6	134.5 ± 5.4	63.0 ± 2.5	131.5 ± 4.6	133.1 ± 3.4	63.7 ± 2.6	128.3 ± 5.2	136.6 ± 4.8

Values are presented as mean ± standard deviation (n = 3). ^1^ Antioxidant capacity of both extracts was measured via the DPPH^•^, ABTS^•+^, and ORAC radical scavenging capacities and is expressed in mg of Trolox equivalents (TE) per 100 g of dry matter (DM). The total quantification of phenolic compounds in both extracts was measured using the Folin–Ciocalteu method and is expressed in mg of gallic acid equivalents (GAE) per 100 g of DM. ^2^ Lipid peroxidation is expressed in nmol TBARS g^−1^ of DM.

**Table 4:** Biometric parameters and whole-body composition of fish fed with the experimental diets for 12 weeks.

	CTRL	VITE	P2	S2	*p*-Value
**Growth performance ^1^**					
Initial body weight (g)	13.5 ± 0.2	13.5 ± 0.03	13.5 ± 0.1	13.5 ± 0.2	0.9
Final body weight (g)	64.5 ± 2.4	59.0 ± 3.4	59.5 ± 4.4	62.3 ± 2.2	0.1
Initial length (cm)	11.2 ± 0.1	11.1 ± 0.1	11.1 ± 0.1	11.1 ± 0.02	0.9
Final length (cm)	17.8 ± 0.1	17.6 ± 0.2	17.4 ± 0.2	17.6 ± 0.2^,^	0.1
K_f_	1.1 ± 0.03	1.1 ± 0.1	1.1 ± 0.05	1.2 ± 0.02	0.3
SGR	1.9 ± 0.04	1.8 ± 0.1	1.8 ± 0.1	1.9 ± 0.04	0.1
DGI	2.0 ± 0.1	1.9 ± 0.1	1.9 ± 0.1	1.9 ± 0.1	0.1
VFI	1.5 ± 0.1	1.5 ± 0.2	1.5 ± 0.1	1.5 ± 0.03	0.9
FCR	1.0 ± 0.04	1.0 ± 0.1	1.0 ± 0.1	1.0 ± 0.03	0.8
PER	1.9 ± 0.1	1.8 ± 0.2	1.9 ± 0.1	1.9 ± 0.1	1.0
**Intake ^2^**					
Dry matter	16.0 ± 0.6	15.8 ± 1.6	15.8 ± 0.6	15.5 ± 0.3	0.9
Protein	8.9 ± 0.4	8.7 ± 0.9	8.8 ± 0.4	8.7 ± 0.2	0.9
Lipids	2.8 ± 0.1	2.7 ± 0.3	2.6 ± 0.1	2.7 ± 0.1	0.8
Gross energy	358.9 ± 14.3	342.2 ± 34.9	359.0 ± 14.4	351.3 ± 7.6	0.6
**Whole-Body Composition ^3^**					
Dry matter	31.0 ± 0.3	31.2 ± 1.7	31.8 ± 0.6	30.6 ± 0.5	0.4
Ash	3.5 ± 0.5	3.9 ± 0.4	4.0 ± 0.4	3.6 ± 0.2	0.3
Protein	17.1 ± 0.3	17.3 ± 1.5	17.7 ± 0.5	16.8 ± 0.3	0.5
Lipids	11.0 ± 0.3	10.9 ± 1.1	11.1 ± 0.3	11.0 ± 0.9	1.0
Gross energy	7.7 ± 0.2	7.5 ± 0.6	7.8 ± 0.4	7.7 ± 0.3	0.8
**Somatic Indexes**					
Viscerosomatic index	6.6 ± 0.4	6.9 ± 0.7	6.8 ± 0.8	7.0 ± 0.5	0.1
Hepatosomatic index	1.1 ± 0.04	1.2 ± 0.2	1.1 ± 0.2	1.1 ± 0.04	0.3

Values are presented as mean ± standard deviation; n = 68 for growth-related parameters and n = 12 for somatic indexes. ^1^ “K_f_” stands for Fulton’s condition index—final, “SGR” stands for specific growth rate, “DGI” stands for daily growth index, “VFI” stands for voluntary feed intake, “FCR” stands for feed conversion ratio, and “PER” stands for protein efficiency ratio. ^2^ Intake is expressed in g of average body weight (ABW) kg^−1^ day, except for gross energy, which is expressed in kJ ABW kg^−1^ day. ^3^ Proximate composition is expressed in % of dry matter (DM), except for energy, which is expressed in kJ g^−1^ DM.

**Table 5:** Plasma metabolites as bioindicators of stress in fish fed with the experimental diets for 12 weeks.

	Non-Stressed				Stressed				*p*-Value		
	CTRL	VITE	P2	S2	CTRL	VITE	P2	S2	Stress	Diet	SxD
**Plasma biomarkers**											
Lactate ^1^	3.3 ± 0.3	3.4 ± 0.2	4.5 ± 0.7	3.4 ± 0.2	6.6 ± 0.8 *	6.6 ± 0.7 *	6.0 ± 0.7 *	6.6 ± 0.6 *	**<0.001**	1.0	0.3
Glucose ^1^	4.2 ± 0.2	4.3 ± 0.3	4.1 ± 0.3	4.6 ± 0.2	7.0 ± 0.3*	6.6 ± 0.2 *	6.8 ± 0.3 *	6.3 ± 0.3 *	**<0.001**	0.9	0.2
Cortisol ^2^	412.0 ± 43.4	394.7 ± 46.4	405.0 ± 23.3	379.8 ± 39.5	480.7 ± 44.9 *	518.8 ± 32.1 *	493.0 ± 57.7 *	425.1 ± 31.9 *	**0.01**	0.5	0.7
Cholesterol ^1^	3.8 ± 0.5	2.1 ± 0.4	3.4 ± 0.5	2.7 ± 0.4	3.6 ± 0.3	3.5 ± 0.4	2.8 ± 0.5	2.8 ± 0.5	0.1	0.4	0.1
Triglycerides ^1^	2.1 ± 0.3	1.3 ± 0.2	2.1 ± 0.4	1.8 ± 0.2	1.8 ± 0.1	1.6 ± 0.2	2.2 ± 0.2	1.7 ± 0.1	0.1	0.8	0.8
NEFA ^1^	0.14 ± 0.01 ^A,B^	0.10 ± 0.01 ^B^	0.16 ± 0.02 ^A^	0.15 ± 0.01 ^A^	0.15 ± 0.01 ^A^	0.14 ± 0.01 ^A,B^	0.14 ± 0.01 ^A,B^	0.13 ± 0.003 ^A,B^	**0.03**	0.4	**0.01**
**Immune parameters ^3^**											
Lysozyme	14.1 ± 4.1	13.1 ± 4.0	11.4 ± 4.0	14.7 ± 4.2	15.4 ± 4.9	13.9 ± 4.2	12.8 ± 3.7	16.1 ± 5.1	0.5	0.3	0.6
Peroxidase	55.5 ± 12.0	72.8 ± 30.4	47.0 ± 8.6	50.9 ± 10.0	64.6 ± 14.0 *	77.6 ± 9.2 *	62.4 ± 10.6 *	68.9 ± 9.1 *	**0.02**	0.7	0.9
ACH50	194.2 ± 28.3 *	217.0 ± 28.8 *	198.9 ± 21.7 *	192.9 ± 24.1 *	160.2 ± 15.0	163.8 ± 31.6	111.7 ± 29.0	108.8 ± 23.2	**0.02**	0.7	0.8

Values are presented as mean ± standard error (n = 12) per dietary treatment. A 2-way ANOVA was performed, where “*” indicates differences between the stressed and non-stressed groups (*p* < 0.05), indicated by numbers in bold, and different uppercase letters (“^A,B^”) represent differences in the interaction between the variables “Stress” and “Diet”. ^1^ Lactate, glucose, cholesterol, triglycerides, and non-esterified fatty acids (NEFAs) are expressed in mmol L^−1^. ^2^ Cortisol is expressed in units mL^−1^. ^3^ Peroxidase is presented in EU mL^−1^, ACH50 is presented in units mL^−1^, and lysozyme is presented in µg mL^−1^.

## Data Availability

Data are contained within the article.

## Abbreviations

The following abbreviations are used in this manuscript:

ABTS^•+^	2,2′-azino-bis(3-ethylbenzothiazoline-6-sulfonic acid)
ABW	Average body weight
ACH50	Complement activity
ANOVA	Analysis of variance
AOAC	The Association of Official Analytical Chemists
BHA	2,3-terc-butil-4-hidroxianisol, E320
BHT	2,6-diterc-butil-*p*-creso, E321
CAT	Catalase
CC	Carbonyl compounds
CDNB	1-chloro-2,4-dinitrobenzene
CF	Crude fat
CIIMAR	Interdisciplinary Centre of Marine and Environmental Research
CP	Crude protein
CTRL	Commercial diet without added antioxidants; negative control
DGI	Daily growth index
DHA	Docosahexaenoic acid
DM	Dry matter
DNA	Deoxyribonucleic acid
DPPH^•^	2,2-diphenyl-1-picrylhydrazyl
EPA	Eicosapentaenoic acid
EU	European Union
FCR	Feed conversion ratio
GAE	Gallic acid equivalents
GPx	Glutathione peroxidase
GR	Glutathione reductase
GSH	Reduced glutathione
GSSG	Oxidized glutathione
GST	Glutathione s-transferase
H_2_O_2_	Hydrogen peroxide
HCl	Hydrochloric acid
K_f_	Fulton’s condition index
LPO	Lipid peroxidation
MDA	Malondialdehyde
MUFA	Monounsaturated fatty acids
NADPH	1-chloro-2,4-dinitrobenzene
NaN_3_	Sodium azide
NaOH	Sodium hydroxide
NEFA	Non-esterified fatty acids
ORAC	Oxygen radical antioxidant capacity
P2	VITE with 2% pineapple peel flour
PER	Protein efficiency ratio
PMS	Post mitochondrial supernatant
PUFA	Polyunsaturated fatty acid
ROS	Reactive oxygen species
S2	VITE with 2% pineapple stem flour
SGR	Specific growth rate
SOD	Superoxide dismutase
TAC	Total antioxidant capacity
TBARS	Thiobarbituric acid reactive substances
TE	Trolox equivalents
TG	Total glutathione
TNB	5-thio-2-nitrobenzoic acid
VFI	Voluntary feed intake
VITE	CTRL diet with 100 mg kg^−1^ of vitamin E; positive control
